# Oral contraceptive use increases risk of inflammatory and coagulatory disorders in women with Polycystic Ovarian Syndrome: An observational study

**DOI:** 10.1038/s41598-019-46644-4

**Published:** 2019-07-15

**Authors:** Saika Manzoor, Mohd Ashraf Ganie, Shajrul Amin, Zaffar A Shah, Imtiyaz A Bhat, S. Douhath Yousuf, Humira Jeelani, Iram A Kawa, Qudsia Fatima, Fouzia Rashid

**Affiliations:** 1 Department of Clinical Biochemistry/Biochemistry, University of Kashmir, Srinagar, J&K India; 2 Department of Endocrinology and Metabolism, Sher-i-Kashmir Institute of Medical Sciences, Srinagar, J&K India; 3 Department of Immunology and Molecular Medicine, Sher-i-Kashmir Institute of Medical Sciences, Srinagar, J&K India

**Keywords:** Metabolomics, Endocrine reproductive disorders

## Abstract

Polycystic ovarian syndrome (PCOS) is a multispectral disorder requiring lifelong management. Its pathophysiology is still being explored which makes its treatment options restrained. Present study explores impact of oral contraceptive mode of treatment on metabolic, hormonal, inflammation and coagulation profile of PCOS women. 50 subjects diagnosed with Rotterdam criteria receiving no drug treatment served as controls whereas 50 subjects receiving only OCPs (Ethinyl estradiol 0.03 mg, Levonorgestrel 0.15 mg) as a mode of treatment at least for six-months served as cases. Ferriman-Gallwey score and hormonal profile improved on OCP treatment. However, parameters like weight, Body mass index, waist-hip ratio, Oral glucose tolerance test, lipid profile, insulin, HOMA-IR, adiponectin, interleukin1β, visfatin, resistin, tissue factor, PT and APTT showed considerable derangements in OCP group. All above parameters are associated with the risk of diabetes mellitus, dyslipidemia, coronary vascular disease, cancers, hypercoagulable state, venous thromboembolism and thrombotic events. Long-term use of OCPs needs to be considered carefully for PCOS patients who are already burdened with associated risk factors. This study was conducted in a region where women do not have much access to high-end screening and diagnostic facilities that further exacerbates their clinical outcomes. Large scale, long-term studies need to be designed to further evaluate safety use of OCPs in PCOS women.

## Introduction

Polycystic ovarian syndrome (PCOS) is a multifactorial disorder involving both reproductive as well as endocrine systems. Based on current Rotterdam criteria the prevalence of PCOS is between 5–10% in reproductive age women^1^. Metabolic issues like inflammation, increased coagulability, visceral obesity, insulin resistance and androgen excess are considered as key features in PCOS^2–4^. Insulin resistance and increased adiposity leads to elevated production of adipocytokines notably adiponectin, visfatin, leptin, resistin, tissue necrosis factor (TNF-α), interleukin-6 (IL-6) and interleukin-1 (IL-1)^5^. These adipocytokines in turn cause the activation of macrophages that leads to production of various proinflammatory mediators^6,7^. Insulin resistance promotes hyperandrogenism in this syndrome^8^ and the interaction of these two conditions with the hemostatic-fibrinolytic systems is of importance and not fully evaluated with reports suggesting that the hyperinsulinemia may alter fibrinolysis^9,10^. Cardiovascular risk markers and atherosclerosis cluster in women with PCOS^11–15^. As a consequence of their hyperandrogenism and insulin-resistant metabolic milieu they are predisposed to prothrombotic state and endothelial dysfunctions^16–21^.

Oral contraceptive pills (OCPs) (estrogen + progestin) are considered as the first-line therapy for PCOS. But there is conflicting data regarding effects of this mode of treatment on metabolism of carbohydrates, lipids, also on insulin, coagulation and inflammation profile in PCOS subjects.

In one of the earlier reports the effect of drug treatment using sample number as 30 OCP users and 30 metformin users indicated OCP has worsening effect on metabolism^22^. In yet another study where 30 women taking OCPs for varying time period were assessed for fibrinogen and platelet count showed derangements in these values^23^.

In present study, our aim was to evaluate PCOS subjects taking OCP treatment versus drug naive PCOS subjects emphasizing evaluation of their metabolic, hormonal, inflammation and coagulation profiles. For this study design we have particularly focused on parameters viz. adiponectin, IL-1β, visfatin, resistin, tissue factor, prothombin time (PT) and activated partial thromboplastin time (APTT) whose brief description is given as follows:-

## Adiponectin

Adiponectin is a chemokine secreted from visceral fat cells and is the only adipo-cytokine that gets down regulated in obesity^24^. It possesses anti-inflammatory, anti-atherogenic and insulin-sensitizing actions^25,26^. Glucose-lowering and anti-inflammatory effects of adiponectin are mediated by the activation and up regulation of the adiponectin receptors^27^.

## Interleukin 1β

Interleukin-1beta (IL-1β) is an effective pro-inflammatory mediator produced by activated macrophages that plays an important role in defense responses to injury and infection^28^. IL-1β is involved in lipid metabolism by regulating insulin levels and has lipase activity under physiological conditions^29^. Further IL-1β is implicated in various diseases like atherosclerosis, chronic heart failure and type 2 diabetes mellitus (T2DM), which are considered as associated risk factors of PCOS^30^.

## Visfatin

Visfatin is an adipocytokine secreted from adipose tissues, hepatocytes, lymphocytes, bone marrow, fetal membranes, liver, muscles and trophoblasts^31^. Visfatin possesses an insulin mimetic effect and binds to insulin receptor, therefore, stimulating glucose uptake in muscle cells and adipocytes while suppressing glucose release from liver cells^32,33^. It also exhibits modulated immune functions and proinflammatory characteristics^34^.

## Resistin

Resistin, an adipokine stimulates SOCS-3 (Suppressor of cytokine-3), which causes inhibition of insulin pathway in the tissues. Moreover, it prevents the phosphorylation of AMPK pathway that is associated with fatty acid oxidation, prevention of cholesterol synthesis and insulin secretion by beta cells of pancreas. Thus elevated levels of resistin down-regulates β-oxidation of fatty acids and glycogen metabolism resulting in dyslipidemia and T2DM consequently^35–37^. Resistin being involved in the processes like inflammation and autoimmune diseases^38^ has been linked to the development of other disease processes like insulin resistance (IR), T2DM, Coronary vascular disease (CVD), atherosclerosis as well which are again considered as risk factors for PCOS^39^.

## Tissue factor

Tissue factor (a trans-membrane lipoprotein) initiates extrinsic pathway which results in the generation of active serine protease i.e., coagulant mediators: FVIIa, FXa & FIIa and thus stimulates fibrin formation. Tissue factor has contribution in producing both hypercoagulability and inflammation that in turn augment each other^40^. This viscous cycle of coagulation-inflammation-thrombosis emerges in the form of various pathologies like diabetes mellitus, cardiovascular diseases, obesity and disseminated intravascular coagulation (DIC) etc.^41,42^.

## PT and APTT

PT gives an indication of the concentration of prothrombin in the blood. Thus PT measures the time taken for fibrin formation or coagulation time through extrinsic pathway. APTT or partial thrombin time (PTT) is a measurement of the intrinsic coagulation pathway and the common pathway. APTT/PTT together measures the time taken for fibrin formation through the intrinsic pathway. PT and APTT are part of lab coagulation screen tests and these are direct reflection of circulating activated coagulation factors present in plasma. One of the indications of hypercoagulable state is decreased APTT values that in turn predict increased risk for future adverse thrombotic and cardiovascular events^43–46^.

## Results

### Basic clinical characteristics of PCOS controls Vs OCP treated cases

PCOS subjects who received no drug treatment served as drug naive PCOS controls (n = 50) while as PCOS subjects who received only OCPs as a mode of treatment served as cases (n = 50). Following parameters like Anthropometry (*BMI*, *Weight*, *waist*-*hip ratio*), Lipid profile (*Cholesterol*, *TG*), *OGTT (Blood glucose fasting*, *Blood glucose 1* *hr*, *Blood glucose 2 hr*) were found deranged in cases as compared to controls (Table 1). Moreover, Hormonal profile i.e., *LH*, *FSH* and *Testosterone* assessed in controls as well as in cases improved in cases i.e., OCP treated group compared to PCOS controls. Insulin profile (*HOMA*-*IR*, *QUICKI*) signified increase in insulin resistance in OCP treated group (Table 2).

**Table 1:** Anthropometric and biochemical parameters of controls (drug naive PCOS) Vs cases (OCP treated PCOS).

Parameters	Mean ± SD (Controls)	Mean ± SD (Cases)	p-value
Menarche	13.10 ± 1.11	12.94 ± 1.11	0.47
Age	22.82 ± 4.83	25.16 ± 4.86	0.018
BMI (kg/m^2^)	23.11 ± 3.71	24.61 ± 3.53	0.041
Weight (kg)	57.44 ± 10.43	60.92 ± 9.34	0.083
Height (cm)	157.53 ± 5.83	157.38 ± 7.34	0.91
Waist (cm)	88.26 ± 9.51	93.46 ± 9.00	0.0060
Hip (cm)	93.22 ± 6.86	97.26 ± 7.07	0.0047
Waist hip ratio	0.95 ± 0.06	0.96 ± 0.06	0.24
FG score	10.12 ± 2.36	7.70 ± 1.25	<0.0001
Blood glucose fasting (mg/dl)	87.50 ± 10.98	89.92 ± 7.84	0.21
Bloodglucose1hr(mg/dl)	120.96 ± 10.70	121.91 ± 7.53	0.61
Bloodglucose2hr(mg/dl)	101.56 ± 9.80	103.06 ± 7.00	0.38
Cholesterol (mg/dl)	167.40 ± 13.60	179.24 ± 19.05	0.0005
Triglycerides (mg/dl)	111.32 ± 16.68	113.58 ± 14.89	0.48
S. Creatinine (mg/dl)	0.88 ± 0.15	0.92 ± 0.20	0.20
S. Uric Acid (mg/dl)	4.37 ± 0.82	4.49 ± 1.06	0.52
Blood Urea (mg/dl)	26.32 ± 4.60	27.71 ± 5.95	0.19
SGPT (IU/L)	20.92 ± 4.06	21.15 ± 3.21	0.76
SGOT (IU/L)	26.09 ± 3.54	27.56 ± 2.43	0.018

^*^Results are expressed as mean and standard deviations (SD). Threshold for statistical significance was set at p < 0.05.

**Table 2:** Hormonal and insulin profile of controls (drug naive PCOS) Vs cases (OCP treated PCOS).

Parameters	Mean ± SD (Controls)	Mean ± SD (Cases)	p-Value
LH (IU/L)	8.99 ± 5.44	3.98 ± 3.18	0.001
FSH (IU/L)	6.50 ± 2.83	6.37 ± 2.88	0.82
LH-FSH Ratio	1.63 ± 1.22	0.74 ± 0.56	0.001
Serum total testosterone (ng/ml)	50.52 ± 17.03	39.34 ± 13.91	0.0005
Fasting Insulin (µIU/ml)	13.31 ± 3.28	15.94 ± 5.05	0.014
HOMA-IR	2.94 ± 1.03	3.49 ± 1.10	0.031
QUICKI	0.33 ± 0.02	0.32 ± 0.01	0.019

^*^Results are expressed as mean and standard deviations (SD). Threshold for statistical significance was set at p < 0.05.

### Evaluation of inflammation profile of PCOS controls Vs OCP treated PCOS cases

The inflammation profile indicates worsening of inflammatory markers in cases compared to controls as depicted: Adiponectin levels were found decreased in cases compared to controls. Statistically significant and negative Pearson’s correlation of Adiponectin was found with *weight* (r = −0.731, p < 0.0001), *waist* (r = −0.656, p < 0.0001), *hip* (r = −0.631, p = 0.0002), *waist*-*hip ratio* (r = −0.443, p = 0.014), *BMI* (r = −0.634, p = 0.0002) and *cholesterol* (r = −0.751, p < 0.0001) (Tables 3 and 4). Multiple regression analysis of Adiponectin shows association with cholesterol and LH-FSH ratio (R square = 0.646, Adjusted R square = 0.620, p < 0.001). Interleukin 1β levels were found elevated in cases compared to controls. Statistically significant and positive Pearson’s correlation of Interleukin 1β was found with *weight* (r = 0.871, p < 0.0001), *waist* (r = 0.774, p < 0.0001), *hip* (r = 0.709, p < 0.0001), *waist*-*hip ratio* (r = 0.580, p < 0.008), *BMI* (r = 0.826, p < 0.0001), and *cholesterol* (r = 0.852, p < 0.0001) (Tables 3 and 4). Multiple regression analysis of Interleukin 1β shows association with age (R square = 0.142, Adjusted R square = 0.111, p = 0.040). Visfatin levels were found elevated in cases compared to controls. Statistically significant Pearson’s correlation of Visfatin was observed with various parameters: *weight* (r = 0.380, p = 0.015), *height* (r = −0.328, p = 0.038), *waist* (r = 0.470, p = 0.002), *hip* (r = 0.317, p = 0.046), *BMI* (r = 0.562, p = 0.002), *cholesterol* (r = 0.391, p = 0.013), *LH* (r = −0.311, p = 0.051) and *FSH* (r = −0.304, p = 0.056) (Tables 3 and 4). Multiple regression analysis of visfatin shows association with BMI, TG and LH-FSH ratio (R square = 0.652, Adjusted R square = 0.612, p < 0.001). Resistin levels were found elevated in cases compared to controls. We did not find any statistically significant Pearson’s correlation of Resistin with the given parameters except with *BMI* (r = 0.334, p = 0.035) (Tables 3 and 4). Multiple regression analysis of Resistin shows association with BMI and SGOT (R square = 0.342, Adjusted R square = 0.293, p = 0.004).

**Table 3:** Comparison of inflammatory and coagulation parameters of controls (drug naive PCOS) Vs cases (OCP treated PCOS).

Parameters	Mean ± SD (Controls)	Mean ± SD (Cases)	p-value
Serum Adiponectin (ng/ml)	6.69 ± 3.26	4.97 ± 2.09	0.0053
Serum Interleukin 1β (pg/ml)	5.11 ± 2.80	10.09 ± 3.07	<0.001
Visfatin (ng/ml)	3.13 ± 0.84	3.30 ± 0.68	0.31
Resistin (ng/ml)	1.84 ± 0.97	2.07 ± 0.87	0.25
Tissue Factor (pg/ml)	98.35 ± 17.23	107.50 ± 13.25	0.0037
Prothrombin Time (sec)	12.27 ± 0.30	11.68 ± 0.30	0.001
Activated Partial Thromo plastin Time (sec)	25.42 ± 1.53	23.48 ± 1.21	0.001

^*^Results are expressed as mean and standard deviations (SD). Threshold for statistical significance was set at p < 0.05.

**Table 4:** Pearson’s correlation of inflammation and coagulation markers with different PCOS diagnostic parameters.

Parameters	Adiponectin	IL-1β	Visfatin	Resistin	Tissue Factor	PT	APTT
Weight	−	+	+				
Waist	−	+	+				
Hip	−	+	+				
Waist-Hip ratio	−	+			+		
Height			−				
FG-score					+		
BMI	−	+	+	+	+		
Cholesterol	−	+	+				
LH			−				
FSH			−				
LH-FSH Ratio					+		

^*^−Sign shows negative Pearson’s correlation; +sign shows positive Pearson’s correlation with different PCOS diagnostic parameters.

### Evaluation of coagulation profile of PCOS controls Vs OCP treated PCOS cases

The coagulation markers assessed in our study were also changed in OCP treated PCOS group in comparison to drug naive group as depicted: Tissue factor levels were increased in cases compared to controls. Significant positive Pearson’s correlation of Tissue Factor was found with various anthropometric parameters like *BMI* (r = 0.34, p = 0.01), *waist*-*hip ratio* (r = 0.34, p = 0.02*)*, *FG*-*score* (r = 0.29, p = 0.04) and *LH*-*FSH ratio* (r = 0.42, p = 0.003) (Tables 3 and 4). Multiple regression analysis of Tissue Factor shows association with *BMI*, *LH*-*FSH ratio* (R Square = 0.267, Adjusted R Square = 0.236, p = 0.001) and *QUICKI* (R Square = 0.160, Adjusted R Square = 0.130, p = 0.029). PT was found decreased in cases compared to controls and its multiple regression analysis shows association with *IR* only (R Square = 0.137, Adjusted R Square = 0.107, p = 0.044) (Tables 3 and 4). APTT was found decreased in cases compared to controls but has not shown correlation with any of the PCOS diagnostic parameters (Tables 3 and 4).

## Discussion

Numerous studies are available which are in favor of considering OCPs as first mode of treatment in PCOS women exhibiting that treatment with OCPs results in decreased free androgens reducing new hair growth and the growth of terminal hair. It also helps to reduce inflammatory acne count by 30–60% with improvement in 50–90% of patients^47^. Estrogen component of OCPs have shown to suppress LH secretion, thus reducing the ovarian androgen production, and also increases sex hormone binding globulin (SHBG) that helps reduce free testosterone. Our results are in agreement with these reports as the present study also showed significant decrease in LH, testosterone and FG-score in OCP treated PCOS women as compared to drug naive PCOS women. There is controversial data available in the literature regarding the impact of OCP use on glucose tolerance and insulin sensitivity in PCOS women. Some reports suggest no change in glucose tolerance and insulin sensitivity while others are suggestive of adverse impact of the drug on these parameters^48–55^. In the present study, we observed the worsening of biochemical parameters like OGTT, lipid profile, fasting insulin, HOMA-IR and QUICKI in OCP treated PCOS women as compared to drug naive PCOS women. This data is suggestive of negative metabolic effects of oral contraceptives in PCOS women as has been reported earlier^49,56^. Our data has also shown statistically significant increase in total cholesterol but insignificant increase in TG in OCP treated PCOS women when compared to drug naive PCOS women. Metabolic disruptions like above for OGTT, lipid and insulin profiles can increase the long-term risk of various metabolic diseases such as T2DM, IR and CVD. Further in our study there was significant increase in waist-hip circumferences and BMI in OCP users as compared to non-users implying obesity with the use of OCPs.

We evaluated various anti and pro-inflammatory cytokines like adiponectin, interleukin 1β, visfatin and resistin in PCOS women and found their levels altered with the use of OCPs. We observed a significant decrease in adiponectin levels in OCP treated PCOS women as compared to drug naive PCOS women. Earlier reports suggest reduced plasma levels of adiponectin are associated with diabetes, obesity, dyslipidemia, hypertension and CVD^57–60^. The results from the present study depict hypoadiponectinaemia in OCP users. Interleukin 1β levels in OCP treated PCOS women were raised as compared to drug naive PCOS women. Increased secretion of IL-1β has been found linked with various autoimmune and auto-inflammatory diseases, also with metabolic deregulations associated with T2DM and impaired beta cell function^61–63^.

Visfatin and resistin in OCP treated PCOS women were both raised as compared to drug naive PCOS women. The circulating levels of visfatin and resistin are found increased in obesity like conditions and their decreased levels are observed in patients using anti-diabetic drugs^34^. Visfatin has been found associated with different types of cancers as it plays an important role in normal cell growth and apoptosis^64^. A link between visfatin induced inflammation and associated malignancies has been reported elsewhere^65^. Visfatin is known as a good marker for endometrial cancer prognosis^66^. Moreover, visfatin levels are raised in various metabolic disorders like obesity, insulin resistance and T2DM, which are well known risk factors for other type of cancers^67^.

Hyperinsulinemia - a key feature in PCOS causes impairment in fibrinolysis resulting in hypofibrinolysis in PCOS women^16,68,69^. Increased fibrinogen contributes to CVD by increasing fibrin formation, plasma viscosity and platelet aggregation^70^. Among OCP users we found significant increase in tissues factor values and statistically significant decrease in PT and APTT values compared to drug naive PCOS women. An earlier study by Zakai NA *et al*., observed that even a mild reduction of APTT could increase the risk of thrombotic events in larger population studies^71^. Hyperandrogenism is another key player of PCOS that along with insulin resistance affects the hemolytic-fibrinolytic system in PCOS women. Although some studies have reported that hyperandrogenism affects coagulation factors and plasma fibrinolytic activity but this aspect needs to be further explored for better understanding. Correlation analysis of inflammatory and coagulatory profile with PCOS diagnostic parameters in the present study using Pearson’s correlation tool showed a general trend of strong association with obesity associated conditions like weight gain, waist, hip, waist-hip ratio, BMI and cholesterol in OCP treated PCOS women. Hence, weight gain is detected as one of the side effects of oral contraceptive treatment that is undesired and can predispose the users to various obesity associated risk conditions.

The present study clearly affirms that on one side the OCP use improves hyperandrogenism and regulates menstrual cyclicity but the other arm i.e., hyperinsulinemia and obesity induced metabolic changes can lead to a mirage of inflammation and coagulation changes which puts the OCP treated PCOS women at risk for various future complications be it T2DM, CVD, VTE, DIC, cancers etc. Decreased risk of endometrial and ovarian cancers has been indicated with the use of OCPs as they are known to regularize the menstrual cyclicity and thus prevents endometrial hyperplasia, however, their long term use can increase the risk of other type of cancers like cervical and breast cancer as has been reported elsewhere^72,73^. Further, it has been reported that OCP use for a period of 10 years doubles the risk of cervical cancer^74^. Our study does not deal directly with oral contraceptive use and the risk of developing cancers, however, our data regarding weight gain, insulin resistance, deranged metabolism and inflammatory markers in OCP treated subjects indicate possibility of increased risk for various cancers in addition to other depicted impediments of this syndrome.

## Conclusion

Our study suggests that OCP as a mode of treatment has indicated efficacy in terms of regularizing menstrual cycles and improving hyperandrogenism thus reducing clinical symptoms like hirsutism, alopecia and acne, but at the same time use of OCPs does contribute to the worsening of disease process. Our data showed worsening of anthropometric, glucose, lipid, insulin, inflammation and coagulation parameters indicating adverse effect of the drug treatment. Thus the interaction of this drug with diverse metabolic pathways leads to increased risk for development of obesity, T2DM, CVD, DIC and VTE. Instead of generalizing OCP as a mode of treatment consideration should be given for high-risk individuals in terms of alternative therapeutic agents and lifestyle modification strategies for better management of disease.

## Materials and Methods

### Study subjects

This observational pilot study (Conducted from Jan. 2014–Mar 2017) was approved by our Institutional Board of Research Studies (BORS), Department of Biochemistry, University Of Kashmir (No: f (BORS-Biological Science, Res/KU/15-10, SIMS 131/1EC-SKIMS/2013-6479) and informed written consent was obtained from the participants (all from Kashmir). Subjects who attended the Endocrinology and Gynecology Clinics, and presented with menstrual disturbances (oligo-/amenorrhea), hyperandrogenism (alopecia, acne and acanthosis), infertility or polycystic ovaries on USG were enrolled. PCOS subjects were selected according to Rotterdam 2003 criteria with all the relevant guidelines strictly followed. Subjects not taking any kind of drug treatment were considered as drug naive PCOS controls (n = 50) and those subjects (n = 50) who took only OCPs (Ethinyl estradiol 0.03 mg, levonorgestrel 0.15 mg) as a mode of treatment for minimum period of six months served as cases. All women had normal renal, hepatic and thyroid functions. Exclusion criteria for the study were ongoing pregnancy, lactation, Non classic adrenal hyperplasia, Cushing’s syndrome, thyroid dysfunction, hyperprolactinaemia, androgen producing tumors, diabetes mellitus, history of coronary artery diseases and coagulation abnormalities. The drug history and smoking status was also evaluated for exclusion criteria.

### Clinical and anthropometric measures

All the study subjects were weighed on an electronic scale in light clothes without shoes. Height without shoes was measured to the nearest centimeter in a standing position with the feet fairly close together. Waist circumference was measured midway between the lower rib margin and iliac crest whereas the hip was measured at the maximum circumference over the buttocks using a non-folded tape. Body mass index (BMI) was calculated as weight (kg) divided by height squared (m²). The clinical assessment included menstrual history and quantitation of hyperandrogenism (i.e., acne vulgaris, alopecia, acanthosis nigricans and hirsutism). Hirsutism assessment has been done using modified FG score. A score of >8 out of 36 has been taken significant and further evidence of PCOS on USG as the presence of 12 or more follicles, measuring 2–9 mm in diameter, in each ovary and/or increased ovarian volume (>10 cm^3^).

### Sample collection

The sampling was done in morning after an overnight fast during early follicular phase (day 2–7) of cycle. The blood samples required for basic biochemical, insulin and hormonal investigations were collected in clot–activator tubes whereas the blood samples required for analysis of adiponectin, IL1- β, visfatin, resistin, tissue factor, PT and APTT were collected in tri-sodium citrate vials.

Investigations and assays

- Basic biochemistry and metabolic profile: Oral glucose tolerance test (OGTT)
  Kidney function test (KFT) - creatinine, uric acid, urea
  Liver function test (LFT) - Serum glutamic pyruvic transaminase (SGPT) and
  Serum glutamic oxaloacetic transaminase (SGOT)
  Lipids –triglyceride, total cholesterol
  Insulin.
- Hormones: Testosterone, Luteinizing hormone (LH), Follicle stimulating hormone (FSH), Thyroid stimulating hormone (TSH), T4 & T3- to rule out thyroid dysfunction, 17-hydroxy progesterone (17-OHP)- to rule out Non classic adrenal hyperplasia and Prolactin (PRL)- to rule out hyperprolactinaemia.
- Inflammation Profile: Adiponectin, IL 1β, Visfatin and Resistin.
- Coagulation Profile: Tissue factor (FIII), Prothrombin time (PT) and Activated partial thromboplastin time (APTT).

Oral glucose tolerance test (OGTT) was performed after 10–14 hour fasting with 75 grams of oral anhydrous glucose load dissolved in 300 ml of water. Blood samples were drawn after every one-hour for OGTT. All the basic biochemical parameters (OGTT, KFT, LFT and Lipid profile) were estimated on semi-automated analyzer (TRANSASIA ERBA CHEM-7) by using ERBA diagnostic Mannheim Gmbh kits. Hormonal analysis (17-OHP, T4, TSH, LH, FSH, Testosterone and PRL) was done by using Chemiluminescence Immunoassays. The sampling for hormonal analysis was done on 2^nd^ to 7^th^ day of the follicular phase of menstrual cycle. Insulin, Adiponectin, Visfatin, Resistin and Tissue factor levels were measured by ELISA on BIORAD analyzer using Raybiotech kits. Interleukin-1β levels were quantified by ELISA on BIORAD analyzer using Boster’s human IL-1β ELISA kit. Levels of PT and APTT were estimated on CA-500 automated analyzer by using SIEMENS kits according to their respective protocols. Quick’s one stage method and Activated partial thromboplastin time method were used for measuring PT and APTT respectively. Insulin resistance and insulin sensitivity were calculated by Homeostatic model assessment of insulin resistance (HOMA-IR) and Quantitative insulin sensitivity check index (QUICKI) respectively^75^.

### Calculations

HOMA-IR was calculated by formula:

$$HOMA-IR=\frac{{\rm{Fasting}}\,{\rm{Insulin}}\,({\rm{\mu }}\mathrm{IU}/{\rm{ml}})\times {\rm{Fasting}}\,{\rm{Glucose}}\,(mg/{\rm{dl}})}{405}$$

QUICKI was calculated by a formula:

$$QUICKI=1/(\mathrm{log}\,fasting\,Insulin+\,\mathrm{log}\,fasting\,Glucose)$$

High HOMA-IR and low QUICKI scores denote Insulin resistance (low Insulin sensitivity).

### Statistics

Statistical analysis was done using SPSS 16.0 version (IBM, Armonk, NY, USA). Parameters like anthropometry, basic biochemistry, hormones and insulin etc measures were compared between cases and controls using two-sampled t-test. Results are expressed as Mean ± SD. Threshold for statistical significance was set at p < 0.05. Pearson’s correlation coefficient (r) and stepwise Multiple regression analysis was used to analyze association among various study variables.
